# Effects of Dietary *Clostridium butyricum* on Growth and Intestinal Mucosal Barrier Functions of Juvenile Channel Catfish (*Ictalurus punctatus*)

**DOI:** 10.3390/microorganisms13051061

**Published:** 2025-05-02

**Authors:** Zihe Guo, Ye Qian, Xiao Peng, Chanxia Qin, Huige Ren, Jingyi Du, Chengrui Huang, Mingzhu Pan, Weihao Ou

**Affiliations:** 1 Key Laboratory of Aquatic Healthy Breeding and Nutrition Regulation of Guangxi Universities, College of Animal Science and Technology, Guangxi University, Nanning 530004, China; 2 Jiangsu Key Laboratory for Exploration and Utilization of Marine Wetland Biological Resources, Yancheng Institute of Technology, Yancheng 224051, China

**Keywords:** *Ictalurus punctatus*, *Clostridium butyricum*, gut microbiota, intestinal metabolome, intestinal health

## Abstract

An 8-week feeding trial was conducted to investigate the effects of dietary *Clostridium butyricum* on the growth and intestinal mucosal barrier functions of juvenile channel catfish (*Ictalurus punctatus*). The diets included the control group feed (CD group) and the treatment group feed (containing 1 × 10^8^ CFU/g *C. butyricum*; CB group). The CB group showed a rising trend in the growth performance. The CB group had significantly higher digestive and antioxidant enzyme activities, and significantly lower malondialdehyde and superoxide anion contents of the intestine. In terms of intestinal mechanical barrier, the CB group showed significantly higher gene expression of intestinal tight junction proteins. With regard to intestinal immune barrier, the CB group displayed significantly lower gene expression of pro-inflammatory factors. Regarding intestinal chemical barrier, the CB group had significantly higher gene expression of *mucin-4*, *β-galactoside-binding lectin*, *lysozyme-c*, and *NK-lysin type 1*. Dietary *C. butyricum* significantly increased the abundance of some beneficial bacteria and increased the levels of some beneficial metabolites in the intestine. Collectively, dietary *C. butyricum* could increase growth, enhance intestinal digestion and antioxidant capacity, strengthen intestinal mucosal barrier, and improve the intestinal metabolism of juvenile channel catfish.

## 1. Introduction

Channel catfish (*Ictalurus punctatus*) is native to North America and is an omnivorous fish species. It has rich nutrition, high meat yield, rapid growth, and strong adaptability, and has become an important farmed fish species [1]. However, the expansion of farming scale, the increase in density, and unscientific management have led to increasingly serious disease problems. Among them, intestinal health issues have become one of the key factors restricting the sustainable development of the channel catfish industry [2]. The intestine plays a key role in the digestion and absorption of nutrients and immune function in aquatic animals. The integrity of the intestinal mucosal barrier is directly related to the intestinal health and life safety of aquatic animals [3,4]. The intestinal mucosal barrier is composed of a mechanical barrier, immune barrier, chemical barrier, and microbial barrier [5]. It is an important defense line to prevent various pathogens from invading the intestine and causing intestinal diseases. Dysbiosis of the gut microbiota can lead to excessive proliferation of harmful microorganisms, inflammation, and disruption of the intestinal mucosal barrier, ultimately resulting in diseases such as enteritis [6,7]. Improving the gut microbiota is helpful to maintaining intestinal microecological balance, preventing pathogen invasion, and protecting intestinal health. Previously, antibiotics were often used in aquaculture to control fish intestinal diseases. But overuse of antibiotics has many side effects, such as damaging fish immune function and health, polluting the environment, and leading to the production of drug-resistant bacteria, thereby affecting food safety and human health. China has banned the addition of antibiotics to feed [8]. Therefore, the application of antibiotic alternatives has received widespread attention, and probiotics are one of the promising antibiotic alternatives.

Probiotics are microorganisms that can improve host health when ingested in sufficient doses and in a live state [9]. Probiotics inhibit the growth of pathogens and reduce their damage to the intestinal mucosal barrier by secreting antibacterial substances (such as bacteriocins and acidic substances), competing with pathogens for adhesion sites and nutrients, increasing the abundance of beneficial bacteria, and enhancing immunity [10,11,12]. Therefore, probiotics have been widely used in aquaculture to promote intestinal health in aquatic animals. *Clostridium butyricum* is a gram-positive and obligate anaerobic probiotic with strong stress resistance [13,14]. Researchers have revealed that *C. butyricum* has the ability to synthesize multiple vitamins, providing essential nutrients for intestinal epithelial cells [15]. In addition, it can also produce enzymes such as lyases and digestive enzymes in the intestine, converting nutrients in feed into usable small molecules and ultimately producing short-chain fatty acids [16]. In aquatic and non-aquatic species, *C. butyricum* can also enhance the mechanical barrier [17,18,19] and chemical barrier of intestinal mucosa [20,21,22], regulate immune response [23,24,25], regulate the balance of the gut microbiota [17,26], improve gut microbial diversity, and increase the abundance of potential beneficial bacteria [19].

Therefore, *C. butyricum* plays an important role in enhancing intestinal mucosal barrier functions to promote intestinal health and boost growth performance. However, there is currently a lack of research on the effects of *C. butyricum* on channel catfish. Hence, this study aims to investigate the effects of adding *C. butyricum* to feed on the growth and intestinal mucosal barrier functions of juvenile channel catfish, so as to provide a reference for improving the growth and intestinal health of fish and promoting the sustainable development of aquaculture.

## 2. Materials and Methods

### 2.1. Feeding Trial and Sample Collection

Juvenile channel catfish used in this experiment were obtained from an aquaculture farm in Nanning, Guangxi, China. The basal diet (Model Number 661; crude protein is 37%, crude fiber is 8%, crude ash is 15%, and crude lipid is 5%) was purchased from Zhanjiang Haida Feed Co., Ltd. (Zhanjiang, China). *C. butyricum* (CGMCC NO.14499) spore powder (1 × 10^10^ CFU g^−1^) was acquired from Qingdao GBW Group Co., Ltd. (Qingdao, China). Fish were acclimated to the rearing environment by feeding on the basal diet for 2 weeks. After 24 h of fasting, fish (approximately 13 g) were randomly divided into the control group (without *C. butyricum*; CD group) and the treatment group (with *C. butyricum*; CB group). Each group consisted of three 400 L plastic tanks (30 fish per tank), with continuous aeration. Fish were fed twice daily (08:00 and 16:00) to apparent satiation. The feeding trial lasted 8 weeks (natural photoperiod). One-third of the water in each tank was replaced daily with dechlorinated and aerated tap water. Residual feeds and feces were promptly removed to prevent water quality deterioration (temperature 26–30 °C; dissolved oxygen > 7.0 mg L^−1^; pH 7.5–8.2; ammonia nitrogen ≤ 0.2 mg L^−1^; nitrite < 0.1 mg L^−1^). For the CB group diet, the proper amount of *C. butyricum* spore powder was completely dissolved in 60 mL sterile ultrapure water and evenly sprayed onto 500 g of the basal diet and mixed manually to achieve a final concentration of 1 × 10^8^ CFU g^−1^, then was dried at 50 °C for 2 h, and finally kept in sealed plastic bags at 4 °C away from light until use. The CD group diet was prepared similarly using an equal volume of sterile ultrapure water without *C. butyricum* supplementation. Fresh diets for both groups were prepared weekly to ensure probiotic viability.

At the end of the feeding trial, fish were fasted for 24 h, anesthetized with an appropriate dose of eugenol, and then weighed and counted. All hindgut samples (without intestinal contents) were collected with sterilized scissors and forceps under aseptic conditions. Hindgut samples from 2 randomly selected fish within each tank were pooled. Each group ultimately included 3 pooled samples for intestinal enzyme activity analysis, 3 pooled samples for intestinal gene expression analysis, 3 pooled samples for intestinal microbiota analysis, and 6 pooled samples for intestinal metabolite analysis. Each pooled sample was placed in sterile, DNase/RNase-free 2 mL centrifuge tubes, then immediately flash-frozen in liquid nitrogen, and finally stored at −80 °C.

The growth performance parameters were calculated using the following formulas:

(1) Weight gain rate (WGR, %) = 100 × (FBW (g) − IBW (g))/IBW (g));

(2) Specific growth rate (SGR, %/day) =100 × [ln (FBW (g)) − ln (IBW (g))]/days;

(3) Feed conversion ratio (FCR) = feed intake (g)/body weight gain (g);

(4) Survival rate (SR, %)= 100 × Nt/Ng.

Notes: Nt is final number of fish; Ng is initial number of fish; IBW is initial body weight; FBW is final body weight.

### 2.2. Intestinal Digestive and Antioxidant Enzyme Activities

The following intestinal parameters were measured using assay kits from Nanjing Jiancheng Bioengineering Institute (Nanjing, China): Total protein content (Model Number A045-4-2), α-amylase activity (Model Number C016-1-1), lipase activity (Model Number A054-1-1), trypsin activity (Model Number A080-2-2), catalase (CAT) activity (Model Number A007-1-1), total superoxide dismutase (T-SOD) activity (Model Number A001-3-2), and malondialdehyde (MDA) content (Model Number A003-1-2). In addition, intestinal glutathione S-transferase (GST) activity (Model Number BC0355) and superoxide anion (O_2_^−^) content (Model Number BC1295) were determined using assay kits from Beijing Solarbio Science & Technology Co., Ltd. (Beijing, China). All experimental procedures were performed in accordance with the manufacturers’ instructions.

### 2.3. Intestinal Mucosal Barrier Functions-Related Gene Expression

The genes selected for real-time PCR included tight junction proteins (*occludin*, *zonula occludens-2* (*zo-2*), *claudin-31*, *claudin-18*, and *claudin-g*) related to intestinal mechanical barrier, anti-inflammatory (*transforming growth factor-β* (*tgf-β*)) and pro-inflammatory factors (*interleukin-1β* (*il-1β*), *tumor necrosis factor-α* (*tnf-α*), *interleukin-6* (*il-6*), *interleukin-17a* (*il-17a*), *interferon-γ1* (*ifn-γ1*), and *interferon-γ2* (*ifn-γ2*)) associated with intestinal immune barrier, as well as genes linked to intestinal chemical barrier (*mucin-4*, *β-galactoside-binding lectin* (*leg*), *lysozyme-c*, and *NK-lysin type 1*).

Total RNA was extracted from intestinal samples using the Trizol method. The extracted RNA was electrophoresed on a 1.2% (*w*/*v*) denaturing agarose gel to assess the integrity followed by determination of concentration with UV-Vis Spectrophotometer Q5000 (Quawell Technology Inc., San Jose, CA, USA). The detection results demonstrated that the quality and quantity of the extracted RNA were satisfactory. Subsequently, the extracted RNA was reverse-transcribed into cDNA using a qPCR-specific reverse transcription kit (Model Number RR047A; Takara Biomedical Technology (Beijing) Co., Ltd., Beijing, China). Real-time PCR was performed using the CFX384 Touch™ Real-Time PCR Detection System (Bio-Rad Laboratories, Inc., Hercules, CA, USA). Real-time PCR reaction system was as follows: cDNA 1 μL, forward primer (10 μM) 0.5 μL, Reverse primer (10 μM) 0.5 μL, 2×RealStar Fast SYBR qPCR mix 10 μL (Model Number A301-10; Genstar, Beijing, China), and ddH_2_O 8 μL. Real-time PCR thermal cycling conditions were as follows: 95 °C 2 min, followed by 40 cycles of “95 °C (15 s), 60 °C (30 s), and 72 °C (30 s)”. The 18S rRNA gene was used as the reference gene. Gene expression levels were analyzed using the 2^−ΔΔCT^ method [27]. The relevant primer sequences are listed in Table S1.

### 2.4. Extraction of Gut Microbial Genomic DNA and 16S rRNA Gene Amplicon Sequencing

The genomic DNA of the gut microbiota from intestinal samples was extracted using the CTAB method [28]. The amplified region is the 16S V4. All PCR mixtures contained 15 µL of Phusion^®^ High-Fidelity PCR Master Mix (New England Biolabs, Inc., Ipswich, United Kingdom), 0.2 µM primers, and 10 ng of genomic DNA template. The initial denaturation was performed at 98 °C for 1 min, followed by 30 cycles of “98 °C (10 s), 50 °C (30 s), and 72 °C (30 s)”, with a final extension at 72 °C for 5 min. The PCR products were then purified using magnetic beads, pooled in equal amounts based on their concentrations, thoroughly mixed, and detected to recover the target bands. Finally, a library was constructed, and the quality of the library was assessed using Qubit and q-PCR. After passing the quality check, the library was sequenced on the NovaSeq 6000 platform using PE250 mode. The raw data of 16S rRNA gene amplicon sequencing had been uploaded to the Sequence Read Archive (SRA) with the accession number PRJNA1233773.

### 2.5. Analysis of 16S rRNA Gene Amplicon Sequencing Data

The sequencing data were demultiplexed based on barcode sequences and PCR amplification primer sequences. After removing the barcode and primer sequences, FLASH [29] was used to assemble the reads for each sample, generating Raw Tags data. Cutadapt software was then employed to match and trim the reverse primer sequences to prevent interference with subsequent analyses. The Raw Tags were rigorously filtered using fastp software (Version 0.23.1) to obtain high-quality tamings data (Clean Tags) [30]. The resulting tag sequences were compared against the Silva database (https://www.arb-silva.de/for16S/18S (accessed on 2 September 2020)) to detect and remove chimeric sequences, yielding the final effective data (Effective Tags) [31]. The Effective Tags were further denoised to obtain Amplicon Sequence Variants (ASVs) and a feature table [32]. Taxonomic annotation was performed using QIIME2 software with the Silva 138.1 database. The data from each sample were normalized to the sample with the lowest sequencing depth. Venn diagram was generated using the VennDiagram function in R. Species abundance statistics were calculated for the top ten species at different taxonomic levels (phylum and genus), and relative abundance distribution histograms were plotted using the SVG function in Perl. Principal co-ordinate analysis (PCoA) was performed and visualized using the ade4 and ggplot2 packages in R (V4.0.3). α-diversity indices, including Chao1, Shannon, Simpson, and Pielou_e, were calculated using QIIME2. Differential microbial communities between groups were analyzed using LDA Effect Size (LEfSe) [33]. Functional prediction was conducted using PICRUSt.

### 2.6. Intestinal LC-MS Untargeted Metabolomics

After precisely weighing 50 ± 5 mg of each intestinal sample, it was placed into a 2 mL centrifuge tube along with a 6 mm grinding bead and 400 µL extraction solution (methanol–water = 4:1 (*v*:*v*)), containing four internal standards (L-2-chlorophenylalanine (0.02 mg/mL), etc.). The sample was homogenized using a cryogenic tissue homogenizer for 6 min (−10 °C, 50 Hz), followed by low-temperature ultrasonic extraction for 30 min (5 °C, 40 KHz). The sample was then incubated at −20 °C for 30 min and centrifuged for 15 min (13,000× *g*, 4 °C). The supernatant was collected for analysis using an LC-MS/MS system UHPLC-Triple TOF 6600 (SCIEX, Foster City, CA, USA). Quality control (QC) samples were prepared by mixing equal volumes of extraction solution from all samples. A QC sample was inserted after every 5–15 analysis samples to assess the stability of the detection process. The raw data were imported into the metabolomics processing software ProgenesisQI v3.0 (Waters Corporation, Milford, MA, USA) for baseline filtering, peak identification, integration, retention time correction, and peak alignment. This resulted in a data matrix containing information on retention time, *m*/*z* ratio, and peak intensity. Feature peak library identification was performed using this software by matching MS and MS/MS spectral information with metabolomics databases. The MS mass error was set to less than 10 ppm, and metabolites were identified based on secondary mass spectrometry matching scores. Key databases included http://www.hmdb.ca/ (accessed on 22 October 2024) and https://metlin.scripps.edu/ (accessed on 22 October 2024), as well as a self-built database. Principal component analysis (PCA) and multivariate statistical analysis of differential metabolites were conducted using ropls (R packages, Version 1.6.2). KEGG compound classification and functional pathway analysis were performed using the KEGG database (kegg_v20230830, Release 2017-05-01). KEGG pathway enrichment, heatmaps, clustering analysis, and correlation analysis were implemented using scipy (Python, Version 1.0.0). Variable importance in projection (VIP) analysis was performed using a combination of ropls (R packages, Version 1.6.2) and scipy (Python, Version 1.0.0).

### 2.7. Statistical Analysis

All data were presented as mean ± SEM. T-tests were performed for statistical analysis. GraphPad Prism 9.0 software (GraphPad, San Diego, CA, USA) was used for plotting. ns indicates *p* > 0.05, * indicates *p* < 0.05, ** indicates *p* < 0.01, *** indicates *p* < 0.001.

## 3. Results

### 3.1. Growth Performance

**Figure 1:**
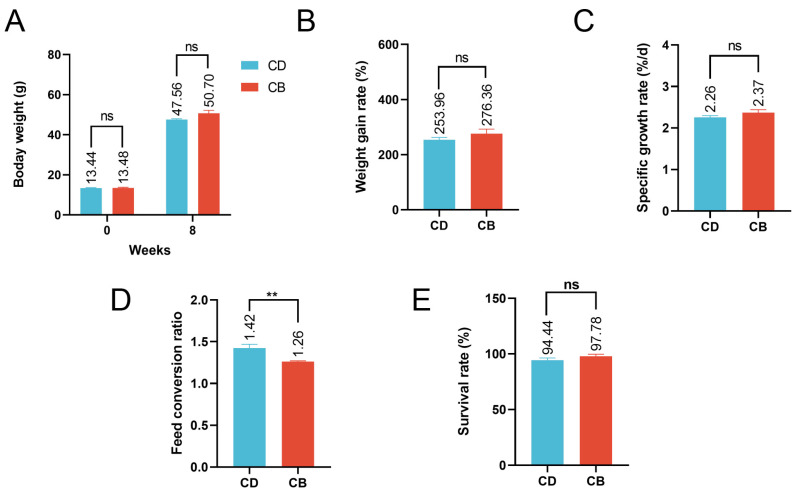
Growth performance of juvenile channel catfish. (**A**) Initial and final body weight; (**B**) Weight gain rate; (**C**) Specific growth rate; (**D**) Feed conversion ratio; (**E**) Survival rate. ns indicates *p* > 0.05, ** indicates *p* < 0.01.

The average final weight, weight gain rate, specific growth rate, and survival rate of the CB group were higher than those of the CD group, but the differences were not significant (*p* > 0.05), while the feed conversion ratio of the CB group was significantly (*p* < 0.01) lower than that of the CD group (Figure 1A–E).

### 3.2. Intestinal Digestion and Antioxidant Capacity

**Figure 2:**
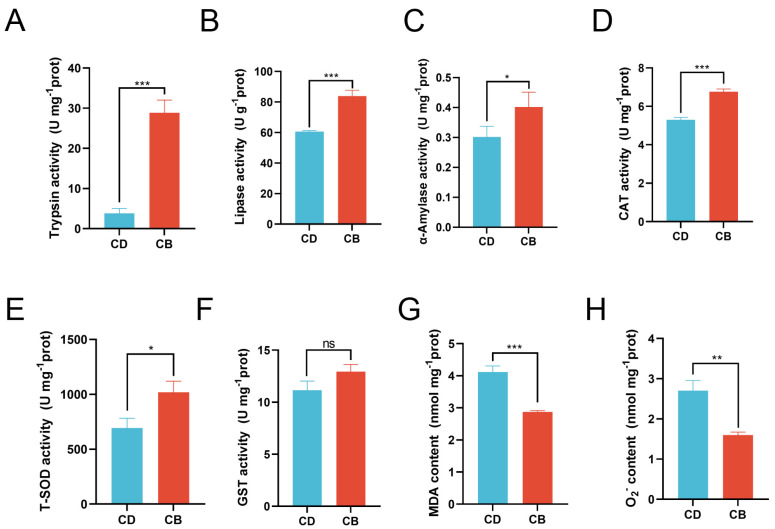
Intestinal digestive and antioxidant enzyme activities of juvenile channel catfish. (**A**) Trypsin activity; (**B**) Lipase activity; (**C**) α-Amylase activity; (**D**) Catalase (CAT) activity; (**E**) Total superoxide dismutase (T-SOD) activity; (**F**) Glutathione S-transferase (GST) activity; (**G**) Malondialdehyde (MDA) content; (**H**) O_2_^−^ content. ns indicates *p* > 0.05, * indicates *p* < 0.05, ** indicates *p* < 0.01, *** indicates *p* < 0.001.

Compared with the CD group, the activities of trypsin (*p* < 0.001), lipase (*p* < 0.001), and α-amylase (*p* < 0.05) were significantly increased in the CB group (Figure 2A–C). Compared with the CD group, the activities of CAT (*p* < 0.001) and T-SOD (*p* < 0.05) in the CB group were significantly increased, while the activity of GST showed no significant difference between the two groups (*p* > 0.05) (Figure 2D–F), and the contents of MDA (*p* < 0.001) and O_2_^−^ (*p* < 0.01) in the CB group were significantly decreased (Figure 2G,H).

### 3.3. Gene Expression Associated with Intestinal Mucosal Barrier Functions

**Figure 3:**
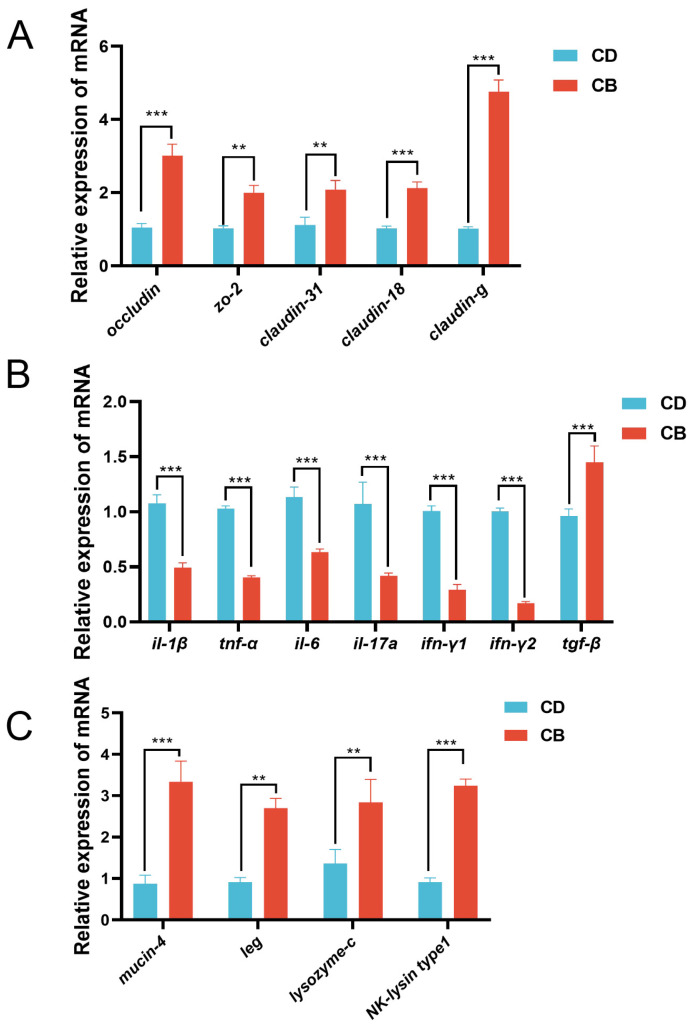
Intestinal mucosal barrier functions-related gene expression of juvenile channel catfish. (**A**) Gene expression of intestinal tight junction proteins; (**B**) Gene expression of intestinal inflammatory cytokines; (**C**) Intestinal chemical barrier-related gene expression. ** indicates *p* < 0.01, *** indicates *p* < 0.001. *zo-2*: *zonula occludens-2*; *il-1β: interleukin-1β*; *tnf-α: tumor necrosis factor-α*; *il-6: interleukin-6*; *il-17a: interleukin-17a*; *ifn-γ1: interferon-γ1*; *ifn-γ2: interferon-γ2*; *tgf-β*: *transforming growth factor-β*; *leg*: *β-galactoside-binding lectin*.

In terms of the intestinal mechanical barrier, compared with the CD group, the CB group had significantly higher gene expression of intestinal tight junction proteins (*occludin* (*p* < 0.001), *zo-2* (*p* < 0.01), *claudin-31* (*p* < 0.01), *claudin-18* (*p* < 0.001), and *claudin-g* (*p* < 0.001)) (Figure 3A). With regard to the intestinal immune barrier, compared with the CD group, the CB group had significantly higher gene expression of anti-inflammatory factor *tgf-β* (*p* < 0.001), and significantly lower gene expression of pro-inflammatory factors (*il-1β* (*p* < 0.001), *tnf-α* (*p* < 0.001), *il-6* (*p* < 0.001), *il-17a* (*p* < 0.001), *ifn-γ1* (*p* < 0.001), and *ifn-γ2* (*p* < 0.001) (Figure 3B). Regarding the intestinal chemical barrier, compared with the CD group, the CB group had significantly higher gene expression of *mucin-4* (*p* < 0.001), *leg* (*p* < 0.01), *lysozyme-c* (*p* < 0.01), and *NK-lysin type 1* (*p* < 0.001) (Figure 3C).

### 3.4. Intestinal Microbiota

We used the high-throughput sequencing technology of the 16S rRNA gene to analyze the diversity, composition, and function of the intestinal microbiota. The α-diversity can reflect microbial diversity, such as richness and evenness, and thus reflect the stability of microecosystems in different groups. This study used the Chao1 index, Shannon index, Simpson index, and Pielou-e index to evaluate the α-diversity between the CD and CB groups. The results showed that there was no significant difference in the four α-diversity indexes between the CD and CB groups (*p* > 0.05) (Figure S1A–D). In addition, each rarefaction curve tended to flatten, indicating that most bacteria had been detected in this study and the sampling depth met the standard to reflect the species diversity in each sample (Figure S1E,F).

**Figure 4:**
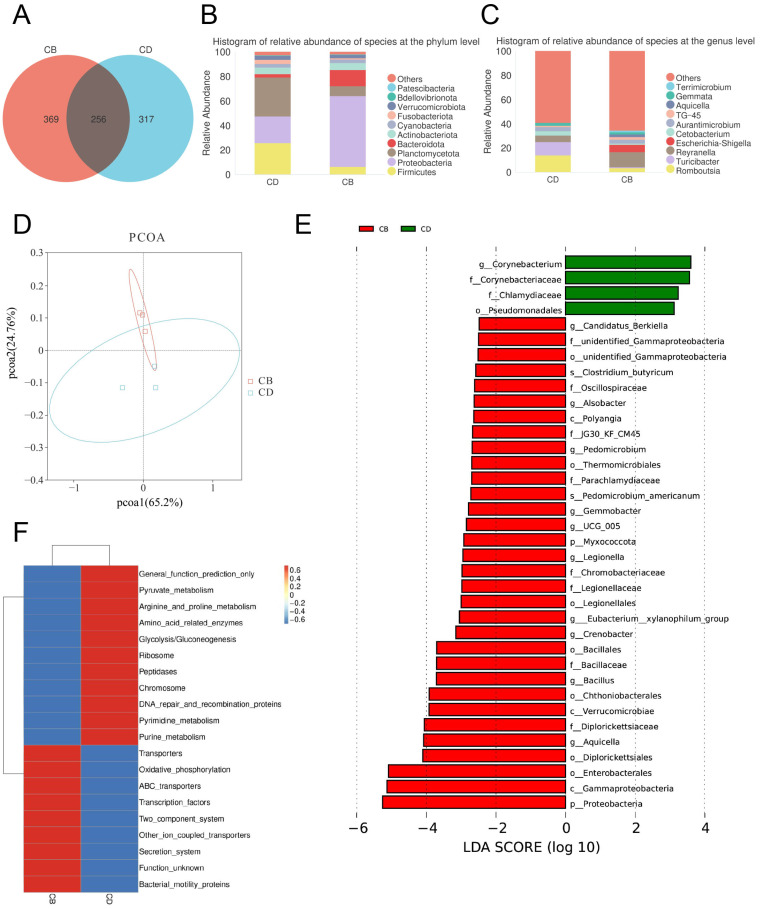
The intestinal microbiota of juvenile channel catfish. (**A**) Venn diagram; (**B**) Top ten bacterial phyla ranked by relative abundance; (**C**) Top ten bacterial genera ranked by relative abundance; (**D**) Principal co-ordinates analysis (PCoA) plot; (**E**) LDA effect size (LEfSe) analysis (bacterium with an LDA score greater than 2 was considered significantly different between the two groups); (**F**) Clustering heatmap of microbial functional abundance based on PICRUSt (the top 20 most significant KEGG level 3 functional categories were selected for visualization, with functional abundance gradients depicted through a color scale ranging from blue (low abundance) to red (high abundance).

The Venn diagram of the intestinal microbiota showed that the number of shared ASVs between the CD and CB groups was 256, the number of unique ASVs in the CD group was 317, and the number of unique ASVs in the CB group was 369, indicating a certain difference in microbial composition between the two groups (Figure 4A). The top ten intestinal bacterial phyla ranked by relative abundance included Patescibacteria, Bdellovibrionota, Verrucomicrobiota, Fusobacteriota, Cyanobacteria, Actinobacteriota, Bacteroidota, Planctomycetota, Proteobacteria, and Firmicutes (Figure 4B). The top ten intestinal bacterial genera ranked by relative abundance included *Terrimicrobium*, *Gemmata*, *Aquicella*, TG-45, *Aurantimicrobium*, *Cetobacterium*, *Escherichia-Shigella*, *Reyranella*, *Turicibacter*, and *Romboutsia* (Figure 4C).

The PCoA plot of the intestinal microbiota showed that the intestinal microbiota of groups CD and CB had clustering, respectively, showing certain differences (Figure 4D). The LEfSe analysis of the intestinal microbiota showed that (Figure 4E) biomarkers of the CD group with statistical difference at the order level included Pseudomcnadales, at the family level included Corynebacteriaceae and Chlamydiaceae, and at the genus level included *Corynebacterium*. The LEfSe analysis of the intestinal microbiota also showed that (Figure 4E) biomarkers of the CB group with statistical difference at the phylum level included Myxococcota and Proteobacteria, at the class level included Polyangia, Verrucomicrobiae, and Gammaproteobacteria, at the order level included unidentified_Gammaproteobacteria, Thermomicrobiales, Legionellales, Bacillales, Chthoniobacterales, Diplorickettsiales, and Enterobacterales, at the family level included unidentified_Gammaproteobacteria, Oscillospiraceae, JG30_KF_CM45, Parachlamydiaceae, Chromobacteriaceae, Legionellaceae, Bacillaceae, and Diplorickettsiaceae, at the genus level included *Candidatus_Berkiella*, *Alsobacter*, *Pedomicrobium*, *Gemmobacter*, UCG_005, *Legionella*, *Eubacterium_xylanophilum_group*, *Crenobacter*, *Bacillus*, and *Aquicella*, and at the species level included *C. butyricum* and *Pedomicrobium americanum*.

The prediction of the intestinal microbiota functions showed that there was a significant difference in the abundance of the top 20 pathways between the CD and CB group. Among them, the abundance of pathways including General function prediction only, Pyruvate metabolism, Arginine and proline metabolism, Amino acid-related enzymes, Glycolysis/Gluconeogenesis, Ribosome, Peptidases, Chromosome, DNA repair and recombination proteins, Pyrimidine metabolism, and Purine metabolism significantly increased in the CD group, while the abundance of pathways including Transporters, Oxidative phosphorylation, ABC transporters, Transcription factors, Two-component system, other ion coupled transporters, Secretion system, Function unknown, and Bacterial motility proteins significantly increased in the CB group (Figure 4F).

### 3.5. LC-MS Untargeted Metabolomics of the Intestine

**Figure 5:**
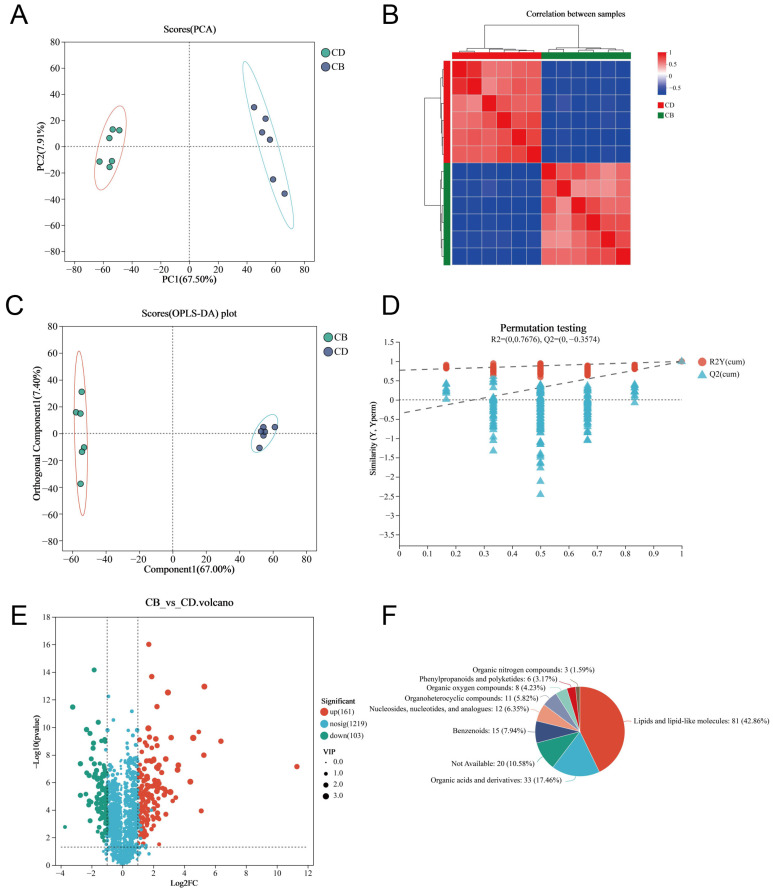
Multivariate analysis of intestinal metabolites of juvenile channel catfish. (**A**) Principal component analysis (PCA) plot; (**B**) Heatmap of Spearman correlation analysis of samples; (**C**) Orthogonal partial least squares discriminant analysis (OPLS-DA) plot; (**D**) Permutation test results of OPLS-DA; (**E**) Volcano plot of differential metabolites (CB vs. CD); (**F**) HMDB compound classification analysis (Superclass-level classification of differential metabolites by HMDB category).

We used LC-MS untargeted metabolomics technology to analyze intestinal metabolites. The PCA plot of the intestinal metabolites showed that the intestinal metabolites of each group had clustering, respectively, and there was no outlier sample (Figure 5A). The heatmap of the Spearman correlation analysis of samples showed clear differentiation between the two groups, indicating high consistency and repeatability among samples within each group (Figure 5B). The Orthogonal partial least squares discriminant analysis (OPLS-DA) plot showed a high degree of metabolite separation between the two groups (Figure 5C). In addition, the permutation test results showed R^2^ = (0, 0.7676) and Q^2^ = (0, −0.3574), indicating that the OPLS-DA model was stable and reliable without overfitting (Figure 5D). VIP values of the OPLS-DA model were extracted and combined with univariate analysis (with VIP > 1, |Log2FC| > 1 and *p* < 0.05 as criteria) to screen intestinal differential metabolites. A total of 264 differential metabolites were screened, including 161 up-regulated differential metabolites and 103 down-regulated differential metabolites in the CB group (Figure 5E). The classification of all differential metabolites by HMDB showed that Lipids and lipid-like molecules, Organic acids and derivatives, as well as Benzenoids were the top three categories with the highest proportion of differential metabolites (Figure 5F).

**Figure 6:**
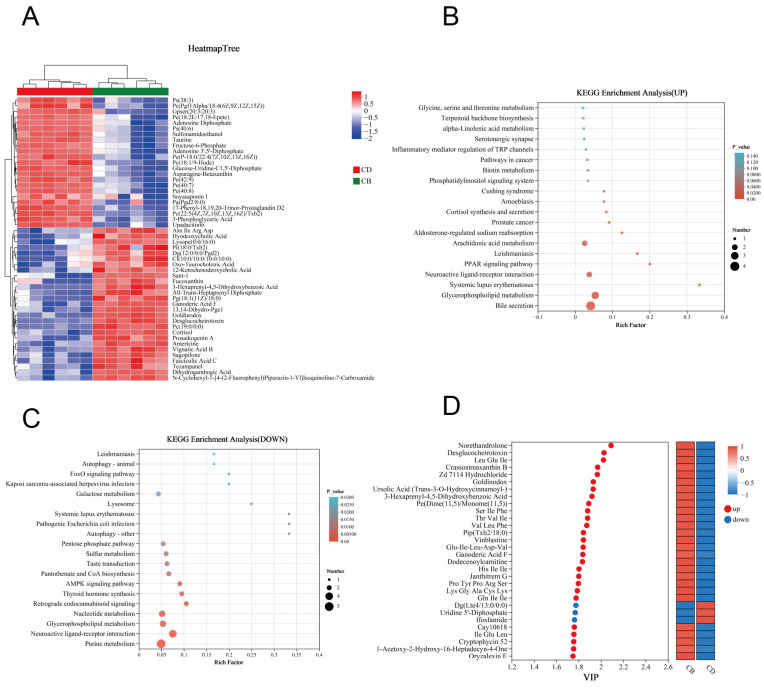
Clustering analysis and KEGG pathway enrichment analysis of intestinal differential metabolites of juvenile channel catfish. (**A**) Clustering heatmap of differential metabolites; (**B**) KEGG annotation analysis of up-regulated differential metabolites; (**C**) KEGG annotation analysis of down-regulated differential metabolites; (**D**) Variable importance in projection (VIP) analysis plot based on the OPLS-DA model (constructed using the top 30 differential metabolites ranked by VIP values).

In order to more intuitively present the variation trends of differential metabolites between the two groups and explore which metabolites had significant changes in different groups, we performed clustering analysis on 264 differential metabolites and selected the top 50 metabolites in abundance for clustering heatmap display. The results showed that the CB and CD groups could be well distinguished by these metabolites (Figure 6A). KEGG enrichment analysis was performed, respectively, for 161 up-regulated differential metabolites and 103 down-regulated differential metabolites in the CB group. The results showed that the up-regulated differential metabolites were enriched in 23 KEGG pathways; for example, Bile secretion, Glycerophospholipid metabolism, PPAR signaling pathway, and Arachidonic acid metabolism were significantly enriched (*p* < 0.05) (Figure 6B). In addition, the down-regulated differential metabolites were enriched in 66 KEGG pathways, of which 27 pathways were significantly enriched; for example, Purine metabolism, Glycerophospholipid metabolism, and Nucleotide metabolism were significantly enriched (*p* < 0.05) (Figure 6C). According to the VIP value (based on the OPLS-DA model) ranking from high to low, the top 30 differential metabolites in VIP values were selected as the key analysis objects (Figure 6D). Among these 30 metabolites, 27 were significantly up-regulated and three were significantly down-regulated in the CB group. Metabolites closely related to intestinal health, such as Gln-Ile-Ile, Ursolic Acid, Ganoderic Acid F, Thr-Val-Ile, CAY10618, and Lys-Gly-Ala-Cys-Lys, were significantly up-regulated.

## 4. Discussion

### 4.1. Dietary C. butyricum Enhanced the Growth Performance and Intestinal Digestive Enzyme Activities of Juvenile Channel Catfish

At present, the benefits of *C. butyricum* as a feed additive are widely recognized. However, there is still a lack of research on the effects of *C. butyricum* on the growth and intestinal mucosal barrier functions of channel catfish. Our research showed that the average final weight, weight gain rate, specific growth rate, and survival rate did not reach statistical significance. However, the consistent upward trend in these parameters of the probiotic treatment group might suggest potential growth-promoting effects of *C. butyricum*. Dietary *C. butyricum* significantly reduced the feed conversion ratio in this study. Previous studies have also shown that *C. butyricum* can promote the growth of fish, for example, largemouth bass (*Micropterus salmoides*) [34]. Our research revealed that *C. butyricum* significantly enhanced the activities of α-amylase, lipase, and trypsin in the intestine of channel catfish, which is similar to previous studies on other aquatic animals [35]. Therefore, *C. butyricum* may promote the digestion and absorption of nutrients in channel catfish by increasing the activities of intestinal digestive enzymes. In addition, *C. butyricum* can convert nutrients in feed into usable small molecules, ultimately generating short-chain fatty acids. Short-chain fatty acids are the main energy source for intestinal epithelial cells, among which butyric acid is preferentially absorbed and utilized by the intestine [36,37]. Therefore, the increase in intestinal digestive enzyme activities and the production of short-chain fatty acids could improve the growth and feed conversion ratio of juvenile channel catfish.

### 4.2. Dietary C. butyricum Elevated Intestinal Antioxidant Enzyme Activities of Juvenile Channel Catfish

Our study found that dietary *C. butyricum* could increase the activities of CAT, T-SOD, and GST, and reduce the production of MDA and O_2_^−^ in the intestine of juvenile channel catfish. Oxidative stress is one of the causes of intestinal barrier damage and the occurrence of intestinal diseases [38]. CAT, T-SOD, and GST are important endogenous antioxidant enzymes that protect the gut from oxidative stress damage [39]. CAT can catalyze the decomposition of hydrogen peroxide into oxygen and water, SOD can catalyze the dismutation of superoxide anion radicals to generate oxygen and hydrogen peroxide, and GST is an important peroxide-decomposing enzyme [40]. MDA is the main product of peroxidation of polyunsaturated fatty acids and is often regarded as a toxic molecule and biomarker of oxidative stress [41]. O_2_^−^ is the precursor of most reactive oxygen species (ROS) and the medium of oxidation chain reactions [42]. A previous study also reported that dietary *C. butyricum* alleviated ammonia-induced intestinal oxidative stress in giant freshwater prawn (*Macrobrachium rosenbergii*) [43]. Dietary *C. butyricum* metabolites increased the intestinal antioxidant capacity of grass crap by activating the Keap1/Nrf2 signaling pathway to augment enzyme activities (such as SOD and CAT) and corresponding gene expression [44]. Therefore, *C. butyricum* may alleviate oxidative stress in the intestine of juvenile channel catfish by increasing antioxidant enzyme activities, thereby promoting intestinal health.

### 4.3. Dietary C. butyricum Increased the Expression of Genes Linked to Intestinal Mucosal Barrier Functions of Juvenile Channel Catfish

Our study indicated that dietary *C. butyricum* could significantly enhance the intestinal mucosal barrier functions of juvenile channel catfish. In terms of the intestinal mechanical barrier, intestinal tight junction proteins are involved in preserving the stability, permeability, and function of the intestinal epithelial barrier [45]. The CB group had significantly higher gene expression of intestinal tight junction proteins (*occludin*, *zo-2*, *claudin-31*, *claudin-18*, and *claudin-g*), indicating that *C. butyricum* had an improving effect on the intestinal mechanical barrier of juvenile channel catfish. Previous studies also pointed out that dietary *C. butyricum* significantly up-regulated the gene expression of the intestinal tight junction proteins of common carp (*Cyprinus carpio*), yellow catfish (*Pelteobagrus fulvidraco*), turbot (*Scophthalmus maximus*), and hybrid grouper (*Epinephelus fuscoguttatus* ♀ × *Epinephelus lanceolatus* ♂) [17,18,19,46].

NF-κB plays an important role in immune response regulation, and its activation can lead to impairment of intestinal tissues by increasing the expression of pro-inflammatory factors and decreasing the expression of anti-inflammatory factors [47]. With regard to the intestinal immune barrier, the CB group had significantly lower gene expression of pro-inflammatory factors (*il-1β*, *tnf-α*, *il-6*, *il-17a*, *ifn-γ1*, and *ifn-γ2*), as well as significantly higher gene expression of anti-inflammatory factor *tgf-β*, indicating a weakened inflammatory response in the gut of juvenile channel catfish. A previous study on tilapia (*Oreochromis niloticus*) has also shown that adding *C. butyricum* to feed could reduce the intestinal inflammatory response [48]. Oxidative stress plays a key role in the initiation and perpetuation of inflammation [49]. *C. butyricum* metabolites have been proven to improve the immune response of grass carp by suppressing the activation of NF-κB and expression of pro-inflammatory factors, accompanied by strengthening antioxidant capacity [44]. The tight junction structure could be affected by pro-inflammatory factors. For example, TNF-α could disrupt tight junction integrity through the regulation of myosin light chain kinase (Mlck) expression via the NF-κB signaling pathway [50]. A previous study in yellow catfish pointed out that the *C. butyricum*-caused down-regulation of *mlck* and *nf-κb* might explain the increased gene expression of tight junction proteins [18].

Regarding the intestinal chemical barrier, the CB group had significantly higher gene expression of *mucin-4*, *leg*, *lysozyme-c*, and *NK-lysin type 1*. *mucin-4* provides a suitable growth environment for beneficial bacteria while preventing the overgrowth of harmful microorganisms, which is crucial for maintaining intestinal homeostasis [51,52]. The *leg* not only regulates immune responses, but also interacts directly with pathogenic bacteria [53]. *NK-lysin type 1* and *lysozyme-c* can exert antimicrobial effects by directly attacking pathogens, thereby maintaining the balance of the gut microbiota [54,55]. A previous study has also shown that *C. butyricum* could enhance the antimicrobial ability of fish intestines [26]. In addition, digestive enzymes are also an important component of the intestinal chemical barrier. As mentioned above, *C. butyricum* enhanced digestive enzyme activities in the intestine of juvenile channel catfish.

These results suggested that *C. butyricum* could enhance the intestinal mucosal barrier functions of juvenile channel catfish, thereby reducing the invasion of pathogens and toxic substances, promoting intestinal health, and reducing the occurrence of diseases. However, it should be noted that a complex regulatory relationship exists between gene expression levels and protein abundance. Up-regulated gene expression levels cannot be directly equated to an increase in the corresponding protein quantity. Future studies incorporating other methods (for example, proteomic and immunohistochemical validation) would be necessary to fully elucidate the functional implications of these gene expression changes.

### 4.4. Dietary C. butyricum Improved the Gut Microbiota of Juvenile Channel Catfish

Our study indicated that dietary *C. butyricum* could improve the gut microbiota of juvenile channel catfish. Although the analysis of microbial α-diversity showed that the two groups had similar overall richness and evenness in their microbiota, the Venn diagram of the microbiota suggested that each group had its own unique ASVs. Moreover, the PCoA plot of the microbiota showed that the microbiota of the two groups had clustering, respectively, showing certain differences. Therefore, *C. butyricum* had a certain impact on the composition of the gut microbiota of juvenile channel catfish. In this study, the top ten intestinal bacterial phyla ranked by relative abundance included Patescibacteria, Bdellovibrionota, Verrucomicrobiota, Fusobacteriota, Cyanobacteria, Actinobacteriota, Bacteroidota, Planctomycetota, Proteobacteria, and Firmicutes, while the top ten intestinal bacterial genera ranked by relative abundance included *Terrimicrobium*, *Gemmata*, *Aquicella*, TG-45, *Aurantimicrobium*, *Cetobacterium*, *Escherichia-Shigella*, *Reyranella*, *Turicibacter*, and *Romboutsia*. Therefore, there are certain differences in the gut microbiota composition of channel catfish in this study compared with previous studies [56]. The reason for these differences may be attributed to the influence of host genetic background, water quality, and diet on the intestinal microbiota of aquatic animals [12,57].

It is worth noting that the LEfSe analysis of the microbiota showed that *C. butyricum* was only enriched in the CB group, indicating that *C. butyricum* had a certain colonization or adhesion ability in the intestine of juvenile channel catfish. *C. butyricum* is known for its production of short-chain fatty acids, especially butyrate, which has been shown to promote the repair and regeneration of intestinal epithelial cells, reduce inflammatory responses, and improve intestinal disease resistance [58]. The LEfSe analysis also showed that dietary *C. butyricum* increased the abundance of other potential beneficial bacteria in the intestine of juvenile channel catfish, such as Oscillospiraceae, *Baillus*, and *Eubacterium*_*xylanophilum*_group. Bacteria of Oscillospiraceae also produce short-chain fatty acids and other beneficial substances that help maintain gut health [59]. Some members of *Baillus* have been proven to have antimicrobial, immunomodulatory, and gut health-promoting effects [60]. Bacteria of *Eubacterium*_*xylanophilum*_group participate in the degradation of dietary fiber and can produce beneficial metabolites, which help maintain microbial diversity and ecological balance in the gut [61]. In contrast, Pseudomonadales and Chlamydiaceae, which were significantly enriched in the control group as shown by LEfSe analysis, are believed to be associated with host health risks. Some bacteria of Pseudomonadales, such as *Pseudomonas*, are pathogenic and can disrupt the balance of the gut microbiota by inhibiting the growth of beneficial microorganisms through toxin production, antibiotic resistance, and competitive dominance [62]. Additionally, members of Chlamydiaceae, particularly *Chlamydia*, are frequently associated with the host immune response and intracellular infections, which may lead to adverse health effects such as immune dysregulation [63]. Previous studies have also demonstrated that dietary *C. butyricum* could improve the gut microbiota of fish [17,19,26].

The functional prediction results of the gut microbiota in this study indicated that the CB group exhibited significant functional enrichment in pathways such as Oxidative phosphorylation, ABC transporters, Transcription factors, Bacterial motility proteins, and Secretion system. Oxidative phosphorylation is the primary pathway for ATP generation in cells, indicating that the gut microbiota of the CB group may possess enhanced energy metabolism capabilities, which are crucial for maintaining the function and proliferation of the gut microbiota [64]. The up-regulation of ABC transporters suggested that the gut microbiota of the CB group exhibited enhanced capabilities in substance transport and cellular material exchange, enabling more efficient nutrient uptake and utilization [65]. Bacterial motility proteins facilitate microbial colonization and movement within the intestinal environment, while the Secretion system may play a pivotal role in mediating interactions between bacteria and their host or other microorganisms [66,67]. These findings provide robust evidence supporting the potential benefits of *C. butyricum* in maintaining intestinal function and health of the host.

### 4.5. Dietary C. butyricum Improved the Intestinal Metabolic Profile of Juvenile Channel Catfish

Our findings demonstrate that dietary *C. butyricum* enhanced the production of beneficial metabolites in the gut of juvenile channel catfish. PCA plot, sample correlation heatmap analysis, and OPLS-DA analysis collectively demonstrated distinct clustering patterns in intestinal metabolite profiles between the two groups. Intriguingly, KEGG pathway enrichment analysis of differential metabolites revealed significant enrichment in pathways including Bile secretion, Glycerophospholipid metabolism, and Neuroactive ligand–receptor interaction. Bile, a digestive fluid synthesized by the liver, is primarily composed of bile acids, cholesterol, and phospholipids. This biochemical complex serves critical physiological roles in emulsifying dietary lipids and facilitating the absorption of lipid-soluble compounds [68]. Gut microbiota-derived metabolites exert regulatory effects on bile acid synthesis and metabolism, thereby modulating host metabolic and immune functions [69]. The significant enrichment of the Glycerophospholipid metabolism pathway suggested altered metabolic dynamics in the intestinal cellular membrane, which may be linked to intestinal barrier functions, immune response, and lipid metabolism. Intestinal health is fundamentally contingent upon the integrity of the intestinal barrier. Specific Glycerophospholipids play a pivotal role not only in maintaining cellular membrane structure but also in facilitating the formation of tight junctions among intestinal epithelial cells. Critically, bidirectional interactions between the gut microbiota and host Glycerophospholipid metabolism may directly modulate intestinal barrier functions and immune response [70]. The Neuroactive ligand–receptor interaction pathway involves bidirectional communication between neurotransmitters and their receptors, ubiquitously present in both neurological and endocrine systems. This pathway participates in diverse physiological processes such as neural signal transmission and endocrine modulation. The intestine serves not merely as an organ for nutrient digestion and absorption, but also functions as a critical neuroendocrine regulatory hub. Neuroactive molecules within the gut can affect host health status by modulating intestinal peristalsis, epithelial secretory functions, and microbial composition [71].

Moreover, among the top 30 differential metabolites ranked by VIP values, 27 were significantly up-regulated (for example, Ursolic Acid, Ganoderic Acid F, and Cay 10618) and three were significantly down-regulated in the CB group. Ursolic Acid demonstrates anti-inflammatory and antioxidant properties that may alleviate intestinal inflammation and protect the mucosal barrier [72]. Ganoderic Acid F exhibits immunomodulatory effects and anti-inflammatory properties, which can improve intestinal immunity and modulate the gut microbiota composition, thereby promoting intestinal health [73]. Cay 10618 is a PPARδ agonist. PPARδ plays a pivotal role in lipid metabolism and inflammation regulation, and its activation alleviates intestinal inflammation [74].

## 5. Conclusions

In conclusion, this study demonstrated that dietary *C. butyricum* significantly reduced the feed conversion ratio, enhanced intestinal digestive and antioxidant enzyme activities, strengthened intestinal mucosal barrier functions, improved the intestinal metabolic profile, and promoted intestinal health, thereby contributing to the growth promotion of juvenile channel catfish. However, the specific mechanisms by which *C. butyricum* exerts its probiotic effects in channel catfish still require further in-depth investigation to provide more robust theoretical support for utilizing *C. butyricum* to enhance the intestinal health and growth performance of fish, and advancing aquaculture practices.

## Data Availability

The original contributions presented in this study are included in the article/Supplementary Material. Further inquiries can be directed to the corresponding author.

## Supplementary Materials

The following supporting information can be downloaded at: https://www.mdpi.com/article/10.3390/microorganisms13051061/s1, (1) Figure S1. α-diversity analysis of the intestinal microbiota of juvenile channel catfish. (A) Chao1 index; (B) Shannon index; (C) Simpson index; (D) Pielou_e index; (E-F) Rarefaction curves demonstrate the variations in Shannon index (E) and Simpson index (F) with increasing sequencing depth for the CD and CB groups (each curve represents an individual sample). (2) Table S1. Primers used in real-time PCR.
