# Supplementary material for: Effects of Dietary Clostridium butyricum on Growth and Intestinal Mucosal Barrier Functions of Juvenile Channel Catfish (Ictalurus punctatus)
Source: Microorganisms. 2025 May 2;13(5):1061. doi: 10.3390/microorganisms13051061 (PMC12114546; doi:10.3390/microorganisms13051061)
Supplement: Supplementary file 1 [file microorganisms-13-01061-s001.zip › Table S1.pdf]

Table S1 Primers used in real-time PCR

Gene	Forward primer (5' – 3')	Reverse primer (5' – 3')	NCBI Reference Sequence
occludin	TCATTTGACGGGGA CTTGTGT	CTGGGCGTGTGAGT GAGAAAC	XM_017467503.3
zo-2	GGACGCGGAAGTGT GACAT	TCGGATTGTCTTTTC CGCCA	XM_017488927.3
claudin-31	CCCATTTTGGCGCGT ATCAG	CCCCAACTGTGATA GGCAGG	NM_001329250.1
claudin-18	AAACCTTGAGGTCC CTCCCT	GCTCCAGTTGTTCA TTGCGG	NM_001329288.1
claudin-g	TATGGTGGTTCTGTC TGGTGC	ACCAAGCATCTGTA GCCCT	NM_001329251.1
il-1β	CGGAGGCTCATTTTA TGTTTATCT	ACAGAGCAGTCACA TATCTCACAA	NM_001200220.1
tnf-α	TATGTAAAGCCGAG GGCGAC	GAGTCAGACCAGCG AGACAC	NM_001200172.1
il-6	CGACCTCCTTACCTG ATGCTT	CTTCATGTAATTGAT CGCCCTG	OM641812.1
il-17a	TGGTTGCTCAGGCT GCTCCTT	ACGCCAGCTTGATG TCATGTTCC	XM_017455432.3
ifn-γ1	ATGTGCGGACTTGTC ATGGT	TCACAAGTGCAGGT GTCCTC	DQ124249.1
ifn-γ2	CCTGGCAAATTGTA CGATGGTC	CGTGCAAGTGATTC TTCAGCG	DQ124250.1
tgf-β	CGTTTCCGCTTCAA GATGGC	GTGCATTTGGTTGC TTTTGCC	XM_017482335.3
mucin-4	CCAGAATATGTACAG GAGCGTT	TGCAGGTGGCAGAA ATAGGAC	KY490705.1
leg	AGCATCATTCCAATA AACCCCC	ACAAGAAGCAAAAC ATCGCTGA	NM_001200929.1
lysozyme-c	GATGGATCAACGGA CTATG	CTGTCTCACTATGGT CTTG	NM_001200789.1
NK-lysin type 1	CTGAGACACAGCTA CTCCCTG	TTGCAGAGACCTCG AAGGAAT	NM_001200208.1
18S	GCAATTATTCCCCAT GAACGAGG	GGACCTCACTAAAC CATCCGA	XR_008395261.1

Notes: *zo-2*: *zonula occludens-2*; *il-1β: interleukin-1β*; *tnf-α: tumor necrosis factor-α*; *il-6: interleukin-6*; *il-17a: interleukin-17a*; *ifn-γ1: interferon-γ1*; *ifn-γ2: interferon-γ2*; *tgf-β*: *transforming growth factor-β*; *leg*: *β-galactoside-binding lectin*; *18S*: *18S ribosomal RNA*. All primer sequences were verified by NCBI Primer-BLAST.
