# Supplementary figures and images for: Effects of Dietary Clostridium butyricum on Growth and Intestinal Mucosal Barrier Functions of Juvenile Channel Catfish (Ictalurus punctatus)
Source: Microorganisms. 2025 May 2;13(5):1061. doi: 10.3390/microorganisms13051061 (PMC12114546; doi:10.3390/microorganisms13051061)

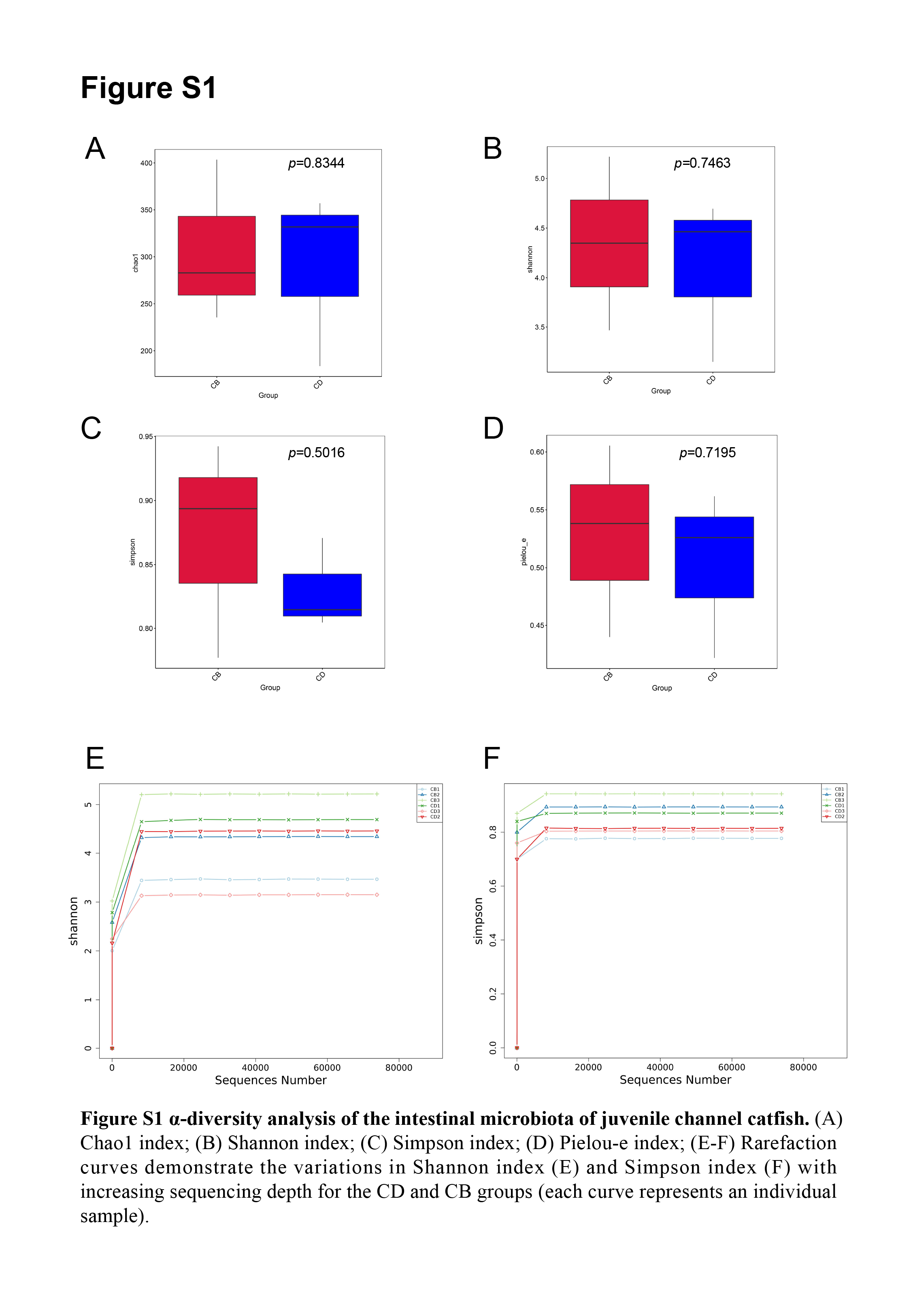

Supplement: Supplementary file 1 [file microorganisms-13-01061-s001.zip › Figure S1.tif]
